# Transcriptome dataset of *Metroxylon sagu* palms from multiple sago plantations in Sarawak

**DOI:** 10.1186/s13104-024-06924-3

**Published:** 2024-09-05

**Authors:** Fifi Hafizzah Pendi, Hasnain Hussain

**Affiliations:** Centre for Sago Research (CoSAR), Faculty of Resource Science and Technology, Universiti Malaysia Sarawak, Kota Samarahan, Sarawak, 94300 Malaysia

**Keywords:** Differentially expressed genes, RNA-sequencing, Stunted growth, Palm, Trunking, Leaf

## Abstract

**Objective:**

Sago palm (*Metroxylon sagu* Rottb.) is one of the most important economic crops abundantly found in Mukah, Sarawak, Malaysia. The robustness of the palm triggered the Sarawak government’s selection as one of the state’s commodity crops, with the opening of several sago palm plantations. However, stunted (non-trunking) palms were reported in several sago palm plantations despite attaining a maturity period of more than ten years after cultivation. Research targeting this problem has been conducted in various fields, yet information on molecular mechanisms is still scarce. This study aimed to determine the genes responsible for sago palm’s normal phenotype (trunking) by attaining leaf transcriptomes from samples of all trunking sago palms from different sago palm plantations.

**Data description:**

The conventional CTAB method was employed in the present investigation to extract total RNA from leaf tissues. Transcriptome sequencing was conducted on the Illumina NovaSeq 6000 platform. Differential expression analysis was performed using the DESeq2 package. A total of 6,119 differentially expressed genes, comprising 4,384 downregulated and 1,735 upregulated genes, were expressed in all three sago palm datasets. The datasets provide insights into the commonly expressed genes among trunking sago palms.

## Objective

Sago palm (*Metroxylon sagu* Rottb.) is a starch-producing palm commonly grown in the Mukah region in Sarawak. The starch of this palm is highly sought after by consumers in Japan and Taiwan for food preparation owing to its superior starch’s gelatinisation behavior [1]. The robust characteristics of this palm set off the Sarawak government’s decision to initiate commercial sago palm plantations to boost the local economy [2]. Nevertheless, stunted palms or non-trunking (no trunking development) sago palms were observed in sago plantations despite reaching the maturity period [3–5]. This condition eliminates the economic value of the affected palms, resulting in a decline in sago starch productivity per hectare of land and, subsequently, instability in sago cultivation among sago planters [6]. Previous research on soil physicochemical and foliar analyses reported nutrient deficiencies in non-trunking sago palms. However, molecular mechanisms to describe this non-trunking are still lacking [7]. Proteomics works suggest that the significantly expressed genes in non-trunking palms were associated with stress factors [4]. Therefore, this article presents experimental data describing the transcriptomic profile of leaf tissue obtained from the trunking sago palms of different plantations. The datasets obtained from this experiment enable the identification of genes of interest and enriched pathways that were expressed in normal (trunking) sago palms growing in different environmental conditions. Ultimately, the datasets provided can supplement the genome sequence to improve sago palm breeding further.

## Data description

The study presents the comparative transcriptome datasets between trunking sago palms from different sago plantations against non-trunking sago palms transcriptome. Trunking samples at *Pelawei Manit* growth stage were selected. Leaf samples obtained from the third frond of the sago bole were used for this experiment. Three biological replications were collected for each collection site. The samples were collected at Dalat Sago Palm Plantation and Sungai Talau Research Station in Mukah as well as Paya Paloh Sago Palm Plantation, in Kota Samarahan. All plantation sites are located in Sarawak, Malaysia [8]. The leaf samples were wiped with 70% ethanol, stored in 50 mL polypropylene tubes, and snap-freeze in liquid nitrogen for transportation. The samples were stored at -80 °C for storage until further use. Total RNA was extracted using a conventional CTAB protocol [9] and subjected to sequencing on Illumina NovaSeq 6000 sequencing platform using paired-end strategy with 2 × 150 bp [10–20]. The non-trunking datasets were obtained from the NCBI SRA repository [21–23] (Table 1). The resultant raw reads from RNA-Seq were subjected to quality control and trimming using an all-in-one FASTQ preprocessor known as *fastp* [24] to yield clean reads [8]. The processed sequencing reads were uploaded to the novoWorx pipeline (Novocraft Technologies Sdn. Bhd.), where the clean reads were aligned against the latest release of published *Metroxylon sagu* genome assembly, Sago_v3 using STAR aligner, sorted using SAMtools, and read counts were obtained using htseq-count. Differentially expressed analysis was performed on htseq-count output using the DESeq2 package following p-adjusted value < 0.05 and log_2_ fold change ≥ 2. Differentially expressed genes yield a total of 6,119 DEGs comprising 4,384 downregulated and 1,735 upregulated genes expressed in all sago palm datasets.

**Table 1:** Overview of data files/data sets

Label	Name of data file/data set	File types (file extension)	Data repository and identifier (DOI or accession number)
Data set 1	Dalat Palm_1 (20 million reads)	FASTQ (.fastq)	NCBI Sequence Read Archive http://identifiers.org/ncbi/insdc.sra:SRX19008328 [10]
Data set 2	Dalat Palm_1 (20 million reads)	FASTQ (.fastq)	NCBI Sequence Read Archive http://identifiers.org/ncbi/insdc.sra:SRX19008329 [11]
Data set 3	Dalat Palm_2 (20 million reads)	FASTQ (.fastq)	NCBI Sequence Read Archive http://identifiers.org/ncbi/insdc.sra:SRX19008331 [12]
Data set 4	Dalat Palm_3 (40 million reads)	FASTQ (.fastq)	NCBI Sequence Read Archive http://identifiers.org/ncbi/insdc.sra:SRX19008332 [13]
Data set 5	Sungai Talau Palm_1 (20 million reads)	FASTQ (.fastq)	NCBI Sequence Read Archive http://identifiers.org/ncbi/insdc.sra:SRX19008333 [14]
Data set 6	Sungai Talau Palm_2 (20 million reads)	FASTQ (.fastq)	NCBI Sequence Read Archive http://identifiers.org/ncbi/insdc.sra:SRX19008334 [15]
Data set 7	Sungai Talau Palm_2 (20 million reads)	FASTQ (.fastq)	NCBI Sequence Read Archive http://identifiers.org/ncbi/insdc.sra:SRX19008335 [16]
Data set 8	Sungai Talau Palm_3 (40 million reads)	FASTQ (.fastq)	NCBI Sequence Read Archive http://identifiers.org/ncbi/insdc.sra:SRX19008336 [17]
Data set 9	Paya Paloh Palm_1 (20 million reads)	FASTQ (.fastq)	NCBI Sequence Read Archive http://identifiers.org/ncbi/insdc.sra:SRX19008337 [19]
Data set 10	Paya Paloh Palm_2 (20 million reads)	FASTQ (.fastq)	NCBI Sequence Read Archive http://identifiers.org/ncbi/insdc.sra:SRX19008338 [18]
Data set 11	Paya Paloh Palm_3 (20 million reads)	FASTQ (.fastq)	NCBI Sequence Read Archive http://identifiers.org/ncbi/insdc.sra:SRX19008330 [21]
Data set 12	Non-trunking_SN7	FASTQ (.fastq)	NCBI Sequence Read Archive http://identifiers.org/ncbi/insdc.sra:SRX13165898 [22]
Data set 13	Non-trunking_SN8	FASTQ (.fastq)	NCBI Sequence Read Archive http://identifiers.org/ncbi/insdc.sra:SRX13165899 [23]
Data set 14	Non-trunking_SN9	FASTQ (.fastq)	NCBI Sequence Read Archive https://identifiers.org/ncbi/insdc.sra:SRX13165900 [24]
Data file 1	Leaf samples’ GPS coordinates	Document file (.docx)	figshare (10.6084/m9.figshare.25991932.v1) [8]
Data file 2	Statistics of the quality and output of the RNA-seq libraries	Document file (.docx)	figshare (10.6084/m9.figshare.25991932.v1) [8]

### Limitations

The present study has its limitations. The selection of the trunking palm at *Pelawei Manit* growth stage was determined through the phenotype of the palm by a sago palm expert from CRAUN Research Sdn Bhd and not by its actual age upon cultivation. The samples obtained are cultivated on different soil types. Dalat sago palm plantation is operated on peat soil whilst both Sungai Talau and Paya Paloh plantations were operated on mineral soil however, no soil samples were collected for this study.

## Data Availability

The data described in this Data Note is accessible at the NCBI Sequence Read Archive under BioProject PRJNA922330: “Metroxylon sagu-Raw sequence reads” (https://www.ncbi.nlm.nih.gov/bioproject/PRJNA922330). The supplementary information described in this Data Note is accessible in figshare repository: 10.6084/m9.figshare.25991932.v1.

## Declarations

### Voucher specimens

The specimen used in this investigation is not deposited in any public herbarium or other public collection. The specimen is currently listed as “Least Concern” by The IUCN Red List of Threatened Species [25].

## Abbreviations

Bp: Base pair
DEGs: Differentially expressed genes
RNA: Ribonucleic acid
